# Comprehensive system biology analysis of microRNA-101-3p regulatory network identifies crucial genes and pathways in hepatocellular carcinoma^☆^

**DOI:** 10.1016/j.jgeb.2025.100471

**Published:** 2025-02-18

**Authors:** Nasim Rahimi-Farsi, Abozar Ghorbani, Negar Mottaghi-Dastjerdi, Taha Shahbazi, Fatemeh Bostanian, Parvin Mohseni, Fateme Yazdani

**Affiliations:** a Department of Biology, University College of Nabi Akram, Tabriz, Iran; b Nuclear Agriculture Research School, Nuclear Science and Technology Research Institute (NSTRI), Karaj, Iran; c Department of Pharmacognosy and Pharmaceutical Biotechnology, School of Pharmacy, Iran University of Medical Sciences, Tehran, Iran; d Razi Hospital, Tehran University of Medical Sciences, Tehran, Iran; e Biochemistry and Biophysics, Tehran University, Tehran, Iran; f Department of Pathobiology, Faculty of Veterinary Medicine, Shahid Bahonar University of Kerman, Kerman, Iran

**Keywords:** Gene network, hsa-miR-101-3p, Hepatocellular carcinoma, Protein-protein interaction

## Abstract

Hepatocellular carcinoma (HCC) is a leading cause of cancer-related mortality worldwide. This study aimed to explore the role of hsa-miR-101-3p in HCC pathogenesis by identifying key genes and pathways. A comprehensive bioinformatics analysis revealed twelve hub genes (ETNK1, BICRA, IL1R1, KDM3A, ARID2, GSK3β, EZH2, NOTCH1, SMARCA4, FOS, CREB1, and CASP3) and highlighted their involvement in crucial oncogenic pathways, including PI3K/Akt, mTOR, MAPK, and TGF-β. Gene expression analysis showed significant overexpression of ETNK1, KDM3A, EZH2, SMARCA4, and CASP3 in HCC tissues, correlating with poorer survival outcomes. Drug screening identified therapeutic candidates, including Tazemetostat for EZH2 and lithium compounds for GSK3β, underscoring their potential for targeted treatment. These findings provide novel insights into the complexity of HCC pathogenesis, suggesting that the identified hub genes could serve as diagnostic or prognostic biomarkers and therapeutic targets. While bioinformatics-driven, this study offers a strong basis for future clinical validation to advance precision medicine in HCC.

## 1 Introduction

MicroRNAs (miRNAs), a class of small, highly conserved RNAs, play a crucial role in regulating transcriptional and post-transcriptional processes by binding to specific mRNAs.^1^ Numerous studies have highlighted the involvement of miRNAs in a wide range of biological processes (BP).^2^ Recent research has emphasized the link between aberrant miRNAs expression levels and critical processes such as cell proliferation, angiogenesis, and metastasis in various human cancers.^3^ miRNAs can act as either oncogenes or tumor suppressors, modulating the expression of their target genes by either enhancing or repressing gene expression.^1^, ^4^ Observing miRNAs dysregulation across different cancer types underscores their potential therapeutic relevance, functioning as oncogenes and tumor suppressors. Among these, hsa-miR-101-3p has attracted considerable attention due to its diverse roles across multiple cancer types and its interactions with key genes that significantly impact cancer progression and metastasis.^3^

Hepatocellular carcinoma (HCC) is currently one of the most common commonly diagnosed cancers, and it ranks as one of the most important leading causes of cancer-related death in men and women, with its incidence continuing to rise.^5^ Recent research efforts have centered on developing drugs that target signaling pathways or genes involved in cancer progression, offering new potential avenues for HCC treatment.^5^ Despite advancements in diagnostics and therapeutics, effective treatment strategies for HCC remain limited, and the overall prognosis is poor, primarily due to late-stage detection.^6^ Therefore, the identification of novel therapeutic targets is crucial to improving survival rates for patients with HCC.

Research has demonstrated miR-101-3p has been shown to significantly promote apoptosis while inhibiting tumor growth, migration, invasion, and metastasis of HCC cells.^3^ Among the confirmed targets of miR-101-3p are Enhancer of Zeste Homolog 2 (EZH2), RAB GTPase 5A (RAB5A), and Dual Specificity Phosphatase 1 (DUSP1).^7^, ^8^ However, given that a single miRNA can influence numerous genes, the precise molecular mechanisms through which miR-101-3p contributes to tumor progression remain unclear. A thorough investigation of the networks of target genes is needed to gain a deeper understanding of the role of miR-101-3p. Therefore, in-depth evaluations of both the clinical relevance and the target proteins associated with miR-101-3p are essential for identifying new opportunities in HCC diagnosis and treatment. This study builds upon and extends previous research on miR-101-3p in HCC by adopting a more comprehensive approach to identify key genes and pathways involved in HCC pathogenesis. Earlier studies typically identified a limited number of miR-101-3p target genes (e.g., 3–5 genes)^9^, ^10^ and often focused solely on individual gene expression or basic pathway analyses. In contrast, our study leverages high-throughput bioinformatics to uncover a broader regulatory network comprising 12 crucial hub genes and their roles in key oncogenic pathways. Furthermore, we go beyond gene identification by incorporating drug screening to identify potential therapeutic compounds, promoter analysis to understand regulatory mechanisms, and survival analysis to assess the prognostic value of hub genes. This integrative approach not only provides a more detailed view of miR-101-3p's regulatory network but also highlights novel therapeutic opportunities, advancing the field's understanding of miR-101-3p in HCC.

High-throughput gene and network analysis methods offer substantial potential for advancing our understanding of cancer biology and developing strategies for early detection and treatment. This research makes significant progress by highlighting dysregulated genes across various pathways, which could be crucial for deciphering the molecular mechanisms underlying cancer progression. Integrating systems biology with systems pharmacology may provide a more comprehensive understanding of these processes. Such an approach facilitates the identification of key gene signatures for personalized medicine and supports the development of precise computational models to predict cellular behavior and tumor dynamics. The use of computational methods and machine learning in the detection and prediction of cancer biomarkers has gained special importance. In particular, the application of deep learning algorithms and classification methods in the identification of molecular patterns and signaling pathways related to miRNAs has opened new horizons in understanding the molecular mechanisms of cancer.^11^ Modern methods based on biosensors and DNA-based diagnostic systems also play an important role in the accurate identification and measurement of biomarkers.^12^ By using multilayer models and algorithms, complex regulatory patterns of miRNAs can be identified and analyzed with higher accuracy.^13^, ^14^, ^15^

In this study, we focus on the therapeutic potential of hsa-miR-101-3p in cancer treatment. Our findings reveal how hsa-miR-101-3p modulates key genes, providing a deeper understanding of the interactions between miRNAs and their targets. This comprehensive analysis identifies 12 crucial genes within the miR-101-3p regulatory network—significantly more than the 3–5 genes typically highlighted in previous bioinformatics studies. Furthermore, unlike earlier studies that primarily emphasized gene identification, we also explored drug screening, identified potential therapeutic compounds, uncovered regulatory motifs, and highlighted survival-associated hub genes. These advancements underscore the potential of hsa-miR-101-3p as a biomarker for HCC treatment strategies.^9^, ^10^

## 2 Materials and methods

### 2.1 Data source

To validate the differences in hsa-miR-101-3p expression between three HCC liver tissues and three normal liver tissues, we obtained relevant data from the NCBI database (GSE98269). Our analysis focused on the expression of hsa-miR-101-3p relative to all miRNAs present in liver cancer, and the initial phase of our study aimed to identify the target genes of hsa-miR-101-3p. For this purpose, we used the miRDB database (https://mirdb.org/cgi-bin/search.cgi). We refined our selection by focusing on genes with a target score of 66 to 100, and the final list was meticulously documented in Excel and included as Supplementary material (Supplementary 1). These identified genes were integral to the subsequent network analysis conducted in this study.

### 2.2 Reestablishing genes, protein-protein interaction (PPI) networks, and application of hub analysis

In this study, the STRING database, which includes both predicted and experimentally validated protein interactions, was utilized to analyze the connections among the genes listed in Supplementary 1. This analysis was performed using the STRING online tool, version 12 (https://string-db.org), resulting in the generation of a PPI network. Only interactions with a medium confidence score of 0.4 or higher were considered. The resulting PPI network was then imported into Cytoscape (version 3.10.1) for further visualization and analysis.

To identify hub proteins within the network, we employed the CytoHubba plugin in Cytoscape. The importance of nodes in the biological network was evaluated using four distinct topological analysis methods: Maximal Clique Centrality (MCC), Degree, Density of Maximum Neighborhood Component (DMNC), and Maximum Neighborhood Component (MNC). The highest-ranking proteins from each method, a total of four, were designated as hub nodes. A subnetwork was subsequently constructed using the CytoHubba plugin to illustrate the interactions between these core nodes.^16^

### 2.3 Cluster analysis

For the cluster analysis of the network, we utilized the STRING version 12 online platform in combination with Cytoscape (version 3.10.1) and the CytoCluster plugin. The protein complex identification algorithm (IPCA) was applied for cluster analysis of the subnetwork, with a threshold set at 0.5 and complex size threshold set at 2 and a shortest path length set at 2. The genes within each identified cluster were further analyzed using STRING version 12 to determine the KEGG pathways associated with these genes.^17^

### 2.4 Analysis of hub genes through gene ontology (GO) and pathway enrichment

Following identifying hub proteins and constructing the subnetwork, we performed an in-depth analysis of the genes linked to the central nodes within the subnetwork. This enhancement study utilized two primary tools: the Kyoto Encyclopedia of Genes and Genomes (KEGG) and GO, which encompass molecular function (MF), cellular components (CC), biological process (BP), and subcellular localization. The STRING online platform facilitated this comprehensive analysis.

### 2.5 Subcellular localization analysis of hub genes

The STRING database version 12 (https://string-db.org/) was used to analyze the subcellular localization of hub genes in protein interaction networks. The main goal of this research was to identify the subcellular localization patterns of hub genes and investigate their relationship with biological functions and disease.

### 2.6 Promoter pattern analysis for central genes

From the Ensembl BioMart web services (https://asia.ensembl.org/info/data/biomart/index.html), we retrieved genomic regions located one kilobase pair upstream of the central genes. We analyzed these sequences using the MEME Suite to identify consistent motifs, focusing on P-value thresholds of less than 0.01. To refine our findings and eliminate redundant motifs while identifying known *cis*-regulatory elements, we utilized the TomTom tool (available at https://meme-suite.org/), referencing the Human JASPAR CORE 2022 (Version 5.5.5) motif database and adhering to the same threshold criteria. To further investigate the potential roles of these motifs, we employed the GoMo tool (Homo sapiens) (available at http://meme-suite.org/tools/gomo). This step provided insights into the functional implications of the identified motifs.

### 2.7 Expression analysis of hub genes

The University of Alabama at Birmingham CANcer data analysis Portal (UALCAN) was utilized to assess the expression levels of the identified hub genes in HCC tissues compared to normal hepatocellular tissues (https://ualcan.path.uab.edu). This portal offers an accessible platform for exploring TCGA-based expression profiles of genes in both tumor and normal tissues.

### 2.8 Survival analysis of hub genes

The prognostic significance of the hub genes was evaluated using Kaplan-Meier overall survival curves for patients with HCC. Survival data were obtained from the UALCAN data analysis portal, which compares the overall survival of patients based on differential expression of the hub genes in HCC. The survival curves were analyzed to assess the impact of high versus low gene expression on patient outcomes.

### 2.9 Screening of the drugs targeting the hub genes

We conducted a drug screening analysis using the UALCAN data analysis portal and its integrated DrugBank link to identify potential therapeutic agents targeting the identified hub genes. This approach enabled us to explore drugs that interact with the hub genes, including their approval status, DrugBank IDs, and known drug targets.

## 3 Results

### 3.1 Reestablishing genes, PPI networks, and application of hub analysis

**Fig. 1:**
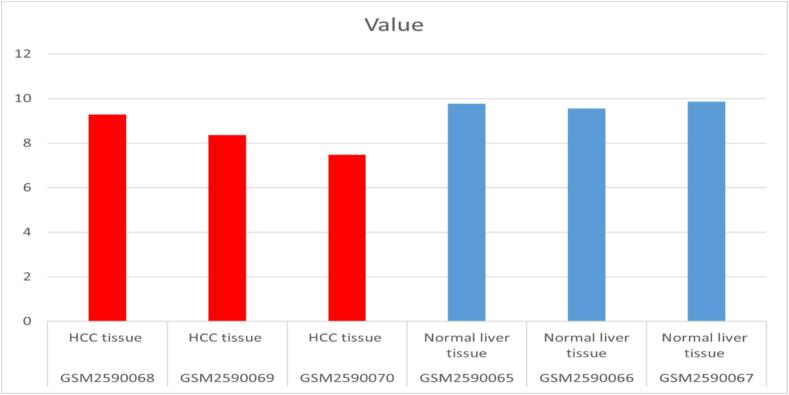
Indicating a decrease in miR-101-3p expression in patients with HCC (p-value = 0.0323, LogFC = −1.381).

Analysis of the GSE98269 dataset provided significant insights into the expression of hsa-miR-101-3p in liver tissue. The data indicated a consistent reduction in hsa-miR-101-3p expression in HCC tissues, supported by graphical representations (P-value = 0.0323, Log Fold Change (LogFC) = −1.381). These findings are illustrated in Fig. 1, which displays the sample values for hsa-miR-101-3p expression across three HCC tissue samples.

**Fig. 2:**
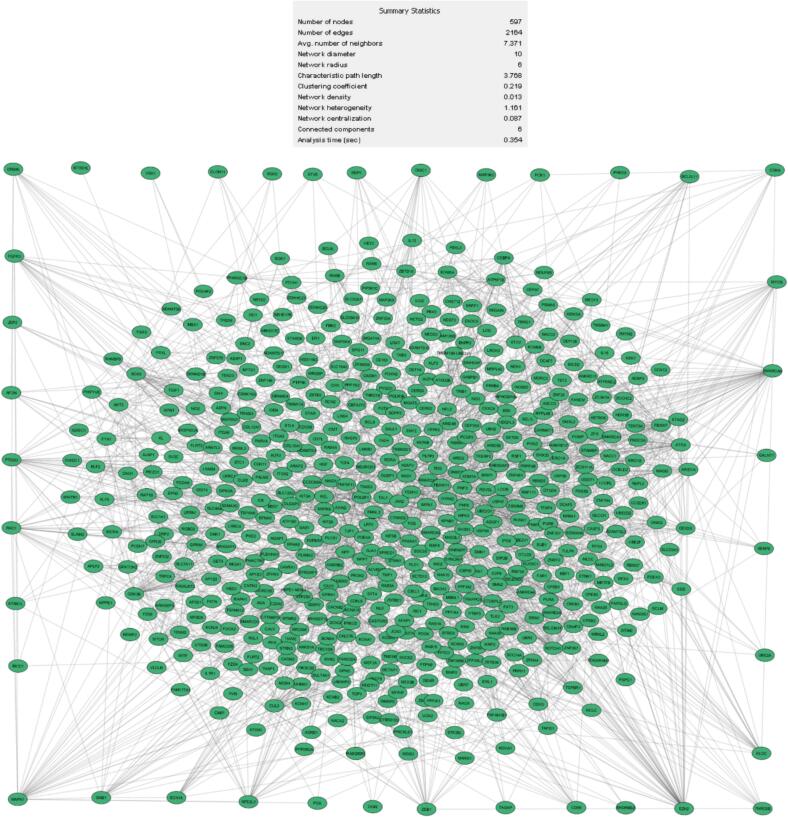
Protein-protein interaction (PPI) network of all expressed genes associated with hsa-miR-101-3p, obtained from the miRDB database. The network consists of 597 nodes and 2164 edges, representing genes and their interactions. The summary statistics of the network, such as the clustering coefficient, network centralization, and density, are shown in the inset, highlighting the complexity and connectivity of the regulatory network. This analysis identifies key regulatory genes and interactions, providing insights into the functional role of hsa-miR-101-3p in cancer biology.

**Fig. 3:**
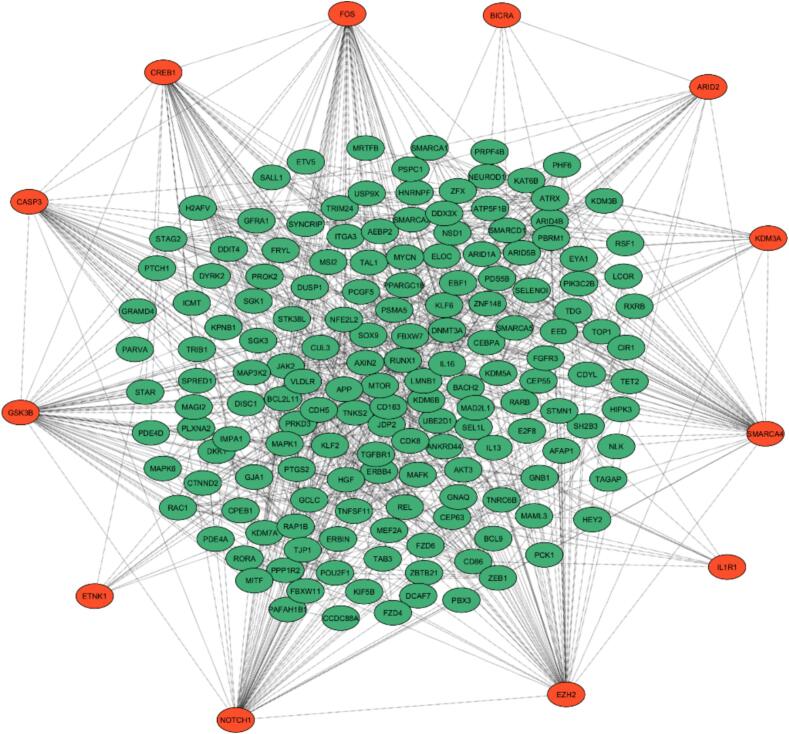
Protein-protein interaction (PPI) network highlighting the 12 hub genes identified using CytoHubba. The hub genes, shown as red nodes (e.g., NOTCH1, CASP3, GSK3B), represent key regulatory elements within the miR-101-3p network. Green nodes depict other interacting genes, and edges represent functional associations. This network illustrates the central role of the hub genes in mediating regulatory interactions, providing insights into their potential as therapeutic targets in cancer biology. (For interpretation of the references to colour in this figure legend, the reader is referred to the web version of this article.)

To further understand the molecular mechanisms underlying HCC, this study focused on identifying differentially expressed genes (DEGs), constructing protein–protein interaction (PPI) networks, and applying hub gene analysis. Using bioinformatics techniques, the HCC DEGs were evaluated compared to normal tissue. Cytoscape software was employed to construct a DEG network comprising 597 nodes and 2164 edges, as shown in Fig. 2. Subsequent hub analysis identified 12 genes that exhibited the highest levels of interaction within the PPI network: ETNK1, BICRA, IL-1R1, KDM3A, ARID2, GSK3β, EZH2, NOTCH1, SMARCA4, FOS, CREB1, and CASP3. These hub genes, which play critical roles in HCC progression, are detailed in Table 1 and Fig. 3.

**Table 1:** List of hub genes identified by using CytoHubba. The hub analysis resulted in the identification of 12 genes with the most interactions with MCC, MNC, DMNC, and Degree.

**Rank cluster**	**Name**	**Description**	**Target Score**
**−**	ETNK1	ETNK1 exhibits heightened ethanolamine phosphotransferase activity, and its overexpression is correlated with a poor prognosis in HCC.	94
**−**	BICRA	BRD4-interacting chromatin remodeling complex-associated protein, also known as Bicaudal C homolog 1, exhibits transcription coactivator activity and possesses histone binding and Tat protein binding capabilities. Additionally, it functions as a tumor suppressor in HCC, with decreased expression levels correlating with advanced disease stages.	100
**−**	IL-1R1	IL-1R1 exhibits diverse binding capabilities, including interactions with cytokine receptors, interleukin-1 receptors, growth factor receptors, signaling receptors, and the tumor necrosis factor receptor superfamily. Additionally, IL-1R1 is involved in protein binding, NAD(P) + nucleosidase activity, NAD + nucleotidase activity, cyclic ADP-ribose generation, and tumor necrosis factor receptor binding. The heightened expression of IL-1R1 is associated with the malignant progression and unfavorable prognosis of HCC.	82
**2**	KDM3A	Also known as lysine demethylase 3A, exhibits various biological activities, including histone H3-methyl-lysine-9 demethylase activity, nuclear receptor binding, nuclear androgen receptor binding, AMP-activated protein kinase activity, cAMP-dependent protein kinase activity, oxidoreductase activity (acting on paired donors, with incorporation or reduction of molecular oxygen), 2-oxoglutarate-dependent dioxygenase activity, enzyme binding, catalytic activity (acting on a protein), histone H3-di/monomethyl-lysine-9 demethylase activity, histone H3-tri/dimethyl-lysine-9 demethylase activity, p53 binding, ubiquitin-protein ligase binding, histone H3-methyl-lysine-36 demethylase activity, transcription coregulator binding, ion binding, protein kinase A regulatory subunit binding, transcription coregulator activity, transition metal ion binding, chromatin binding, transcription regulator activity, E-box binding, catalytic activity, and protein binding. The upregulation of KDM3A has been linked to the promotion of HCC progression and stemness, and it is associated with EMT.	78
**1–2**	ARID2	ARID2 exhibits various molecular activities, including transcription coregulator and coactivator functions, chromatin binding, histone binding, Tat protein binding, RNA polymerase II-specific DNA-binding transcription factor binding, nuclear receptor binding, and lysine-acetylated histone binding. Additionally, it demonstrates DNA binding capabilities. Notably, ARID2 interacts with p53 and acts as a tumor suppressor in liver cancer. Its inactivation has been linked to hepatocarcinogenesis.	71
**3–4**	GSK3β	GSK3β exhibits various molecular interactions, including binding with β-catenin, Protein kinase, I-SMAD, Ubiquitin protein ligase, and Dynactin. It functions as a molecular adaptor. In HCC, GSK3β expression is notably reduced in both tissues and cell lines. The suppression of GSK3β expression is associated with increased tumor growth and metastasis.	84
**1–2-3**	EZH2	EZH2 exhibits various biological activities, including chromatin binding, promoter-specific chromatin binding, methyltransferase activity, DNA (cytosine-5-) methyltransferase activity, transcription corepressor binding, protein methyltransferase activity, DNA binding, nucleic acid binding, S-adenosylmethionine-dependent methyltransferase activity, transcription corepressor activity, histone methyltransferase activity, sequence-specific DNA binding, histone binding, transcription factor binding, RNA polymerase II *cis*-regulatory region sequence-specific DNA binding, and RNA polymerase II core promoter sequence-specific DNA binding. Overexpression of EZH2 has been linked to the promotion of growth, migration, and invasion of HCC cells, thereby contributing to the progression of this malignancy. Moreover, its elevated expression is associated with a poor prognosis in affected individuals.	99
**1–2-3–4**	NOTCH1	NOTCH1 operates as a receptor for membrane-bound ligands, specifically Jagged-1 (JAG1), Jagged-2 (JAG2), and Delta-1 (DLL1), playing a crucial role in the regulation of cell fate determination. It exhibits activities such as Notch binding, calcium ion binding, signaling receptor binding, and transcription coactivator activity. As Notch receptor 1, its upregulation has been associated with heightened migration and invasion of HCC cells, negatively impacting the prognosis of affected individuals.	87
**1–2**	SMARCA4	Identified as SWI/SNF related, matrix-associated, actin-dependent regulator of chromatin, subfamily A, member 4 (SMARCA4), is associated with various cellular functions including transcription coregulator activity, transcription coactivator activity, histone binding, lysine-acetylated histone binding, nuclear receptor binding, RNA polymerase II-specific DNA-binding transcription factor binding, p53 binding, Tat protein binding, protein N-terminus binding, DNA binding, and transcription corepressor activity.	95
**3–4**	FOS	A proto-oncogene and a subunit of the AP-1 transcription factor, possesses DNA-binding transcription activator activity specific to RNA polymerase II, RNA polymerase II *cis*-regulatory region sequence-specific DNA binding, RNA polymerase II-specific DNA-binding transcription factor binding, chromatin binding, transcription coregulator binding, cAMP response element binding, and R-SMAD binding. The proto-oncogene FOS has been implicated in hepatocarcinogenesis, and its upregulation is associated with an unfavorable prognosis in patients with HCC.	94
**3–4**	CREB1	CREB1 serves as a DNA-binding transcription factor involved in various molecular processes. It exhibits RNA polymerase II-specific DNA-binding transcription factor binding, chromatin binding, p53 binding, DNA binding, protein binding, cAMP response element binding protein binding, chromatin DNA binding, ubiquitin-protein ligase binding, transcription regulator activity, transcription co-regulator binding, cAMP response element binding, DNA-binding transcription activator activity, RNA polymerase II-specific activity, R-SMAD binding, and histone acetyltransferase activity. The upregulation of CREB1 has been associated with intrahepatic metastasis in HCC, suggesting its involvement in the progression of this disease. Additionally, elevated CREB1 expression is indicative of a poor prognosis in affected individuals.	67
**3–4**	CASP3	CASP3, a cysteine-type endopeptidase, displays various biological functions such as regulator and inhibitor activities involved in the apoptotic process, enzyme inhibitor and regulator activities, cysteine-type endopeptidase activity associated with apoptotic signaling pathways and the execution phase of apoptosis, peptidase regulator activity, catalytic activity acting on a protein, ubiquitin-protein ligase activity, catalytic activity, cyclin-dependent protein serine/threonine kinase inhibitor activity, enzyme binding, death receptor binding, and enzyme activator activity. Reduced expression of CASP3 has been correlated with intrahepatic metastasis and advanced stages of HCC. This downregulation is indicative of a poor prognosis in affected individuals.	68

### 3.2 GO and KEGG enrichment analysis of hub genes

**Fig. 4:**
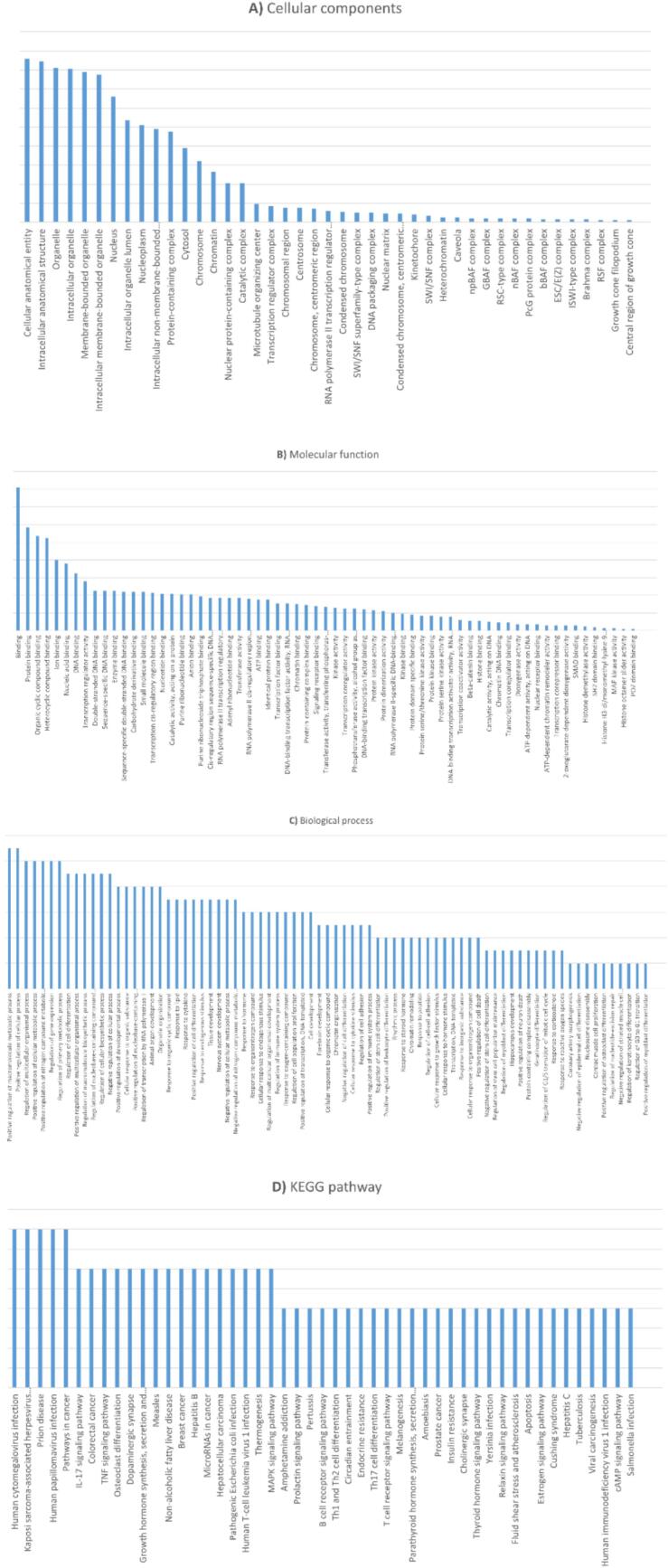
Gene Ontology (GO) and KEGG pathway enrichment analysis of genes associated with hsa-miR-101-3p. The results are categorized into (A) Cellular Components, (B) Molecular Functions, (C) Biological Processes, and (D) KEGG Pathways. The bar charts depict the number of genes enriched in each category, like cellular components involved in binding and regulatory activities, including protein binding, nucleic acid binding, and transcription regulator activity. It highlights the interactions and functions within the cellular environment. Molecular functions and cellular anatomical entities, such as organelles, intracellular structures, and protein-containing complexes. Pathways and Regulatory Processes Chart outlines various viral infections, cancer pathways, and signaling pathways, alongside their corresponding regulatory processes. It highlights the complex interactions between different biological pathways and their roles in cellular and organismal regulation, including metabolic processes, immune responses, and developmental regulation. Also, enrichments provide insights into the functional roles of hsa-miR-101-3p and its potential therapeutic significance in cancer biology.

GO analysis is a widely recognized method for understanding the biological characteristics of extensive genome and transcriptome datasets. It encompasses three primary components: MF, CC, and BP. The results from the GO and pathway enrichment analyses are visually represented in Fig. 4. This analysis, conducted using the STRING online platform, specifically addressed the identified genes' MF, CC, and BP aspects.

The GO terms associated with MF revealed that over 50 % of the genes exhibited significant enrichment in various binding activities. These included binding cellular, protein binding, organic cyclic compound binding, heterocyclic compound binding, ion binding, nucleic acid binding, DNA binding, transcription regulator activity, enzyme binding, as illustrated in Fig. 4.

In the context of CC, the predominant GO terms, representing at least 50 % of the identified genes, included intracellular anatomical structures, organelles, intracellular organelles, membrane-bounded organelles, intracellular membrane-bounded organelles, nuclei, intracellular organelle lumens, nucleoplasm, intracellular non-membrane-bounded organelles, protein-containing complexes, cytosol, chromosomes, chromatin, and nuclear protein-containing complexes.

Regarding BP, the main GO terms (also representing ≥50 % of the genes) encompassed the posetive and negative regulation of biological processes, primary metabolic processes, nitrogen compound metabolic processes, regulation of gene expression, responses to stimuli. Additionally, terms such as regulation of transcription by RNA polymerase II, positive regulation of developmental process, regulation of cellular biosynthetic process, nervous system development, were also highlighted. These findings offer valuable insights into the functional roles and interactions of the identified genes within these biological processes, molecular functions, and cellular components. These findings offer valuable insights into the functional roles and interactions of the identified genes within these biological processes, molecular functions, and cellular components.

In this study, we performed an enrichment analysis utilizing the KEGG database, specifically focusing on the hub genes within the subnetwork. The analysis concentrated on 597 nodes selected to highlight the complex connections and networks of hub genes, which play a pivotal role in systems biology and their integration within the HCC gene framework. This methodology enables a comprehensive exploration of transcriptomic data, emphasizing the modeling of gene networks and the detailed examination of biological pathways using various bioinformatics tools. The KEGG database is a critical resource, linking specific pathways to sets of differentially expressed genes (DEGs) and connecting omics information with a broader spectrum of functional data. This integration is illustrated in Fig. 4, which enhances our understanding of the functional implications of the identified genes in HCC. The KEGG enrichment analysis identified several core pathways that play a significant role in HCC. These include cancer-related pathways, such as the pathways in cancer and miRNAs in cancer, alongside the specific HCC pathway and human cytomegalovirus infection, human papillomavirus (HPV) infection, and pathogenic *Escherichia coli* infection, human T-cell leukemia virus type 1 infection, thermogenesis. Notably, critical signaling pathways such as the PI3K-Akt and MAPK signaling pathways were also highlighted. Moreover, the analysis revealed pathways associated with neurodegenerative diseases, including Alzheimer’s disease, and prion disease. The Human papillomavirus infection pathway is particularly interesting, which involves At least 10 % of the identified genes (Fig. 4). This comprehensive overview of enriched pathways provides valuable insights into the molecular processes and interactions associated with.

### 3.3 Subcellular localization analysis of the hub genes

Subcellular localization analysis provided valuable insights into the expression of hub genes. Notably, the hub genes were found to be represented in various complexes, including protein-containing complexes, the nuclear lumen, catalytic complexes, nucleoplasm, chromatin, DNA packaging complexes, SWI/SNF complexes, SC-type complexes, and GBAF complexes (Table 2). This information provides critical insights into genes' spatial organization and roles within cellular structures, emphasizing their potential functional relevance in HCC.

**Table 2:** Subcellular localization.

Term Description	Hub Genes in the Network
Protein-Containing Complex	FOS, CASP3, EZH2, GSK3β, ARID2, SMARCA4, BICRA, IL-1R1, KDM3A, CREB1, NOTCH1
Nuclear Lumen	FOS, CASP3, EZH2, GSK3β, ARID2, SMARCA4, BICRA, KDM3A, CREB1, NOTCH1
Catalytic Complex	CASP3, EZH2, ARID2, SMARCA4, BICRA, KDM3A
Nucleoplasm	FOS, CASP3, EZH2, GSK3β, SMARCA4, KDM3A, CREB1, NOTCH1
Chromatin	EZH2, ARID2, SMARCA4, BICRA, CREB1
DNA Packaging Complex	ARID2, SMARCA4, BICRA
SWI/SNF Complex	ARID2, SMARCA4, BICRA
RSC-Type Complex	ARID2, SMARCA4
GBAF Complex	SMARCA4, BICRA

### 3.4 Network cluster analysis

Analyzing clusters within biological networks is essential for understanding functional units and predicting network markers and protein groupings. Effective visualization of clustering results is crucial for presenting the structure of the biological network. In our study, we utilized CytoCluster, a versatile clustering tool incorporating six distinct algorithms. The selection of the appropriate clustering method is contingent upon the specific needs and objectives of the user.

For this analysis, we employed the IPCA algorithm, a density-oriented method adept at identifying dense clusters within protein interaction networks. The IPCA algorithm assigns a weight to each connection by evaluating the mutual neighbors associated with its nodes. In contrast, the weight of a node is determined by aggregating the weights of its corresponding connections. This methodology facilitates a comprehensive exploration of clusters within the biological network, enhancing our understanding of the functional relationships among genes and proteins.^17^

To be recognized as significant, a node must exhibit a higher weight. Initially, a seed node is identified as the core of an individual cluster. The IPCA algorithm then expands this cluster by iteratively incorporating neighboring vertices, considering each nodes priority in this process. The decision to include a node in a cluster is influenced by two critical factors: the interaction probability and the shortest path between the node in question and those already included in the cluster.^17^, ^18^

The cluster analysis of the subnetwork revealed a total of 439 clusters. This analysis identified twelve clusters, with clusters ranked 1 to 4 selected for discussion in this paper, as detailed in Table 3. The pathways consistently identified across all four cluster rankings include those previously validated for their associations with HCC, such as miRNAs in Cancer, Pathways in Cancer, the specific HCC pathway, and Thermogenesis. Additionally, several pathways related to signaling and various cancer types were also recognized.

**Table 3:** Based on information supplied using the CytoCluster App, a summary of the clusters (Ranks 1 through 4) emerged from the cluster analysis of the subnetwork of the overexpressed genes in liver cancer together with their known neighbors.

**Cluster Rank**	**Nodes**	**Edges**	**Pathways**
**1**	20	126	HCC and thermogenesis
**2**	20	121	HCC and thermogenesis
**3**	19	109	Colorectal cancer, Prolactin signaling pathway, Prostate cancer, miRNAs in cancer, Endocrine resistance, Pancreatic cancer, HCC, Gastric cancer, Breast cancer, Endometrial cancer, Melanogenesis, Renal cell carcinoma, Central carbon metabolism in cancer, Platinum drug resistance, Human cytomegalovirus infection, Apoptosis, TGF-β signaling pathway, Pathways in cancer, small cell lung cancer, signaling pathways regulating Proteoglycans in cancer, mTOR signaling pathway, PI3K-Akt signaling pathway
**4**	19	111	Colorectal cancer, Prostate cancer, Breast cancer, miRNAs in cancer, Endometrial cancer, Choline metabolism in cancer, Melanogenesis, HCC, Non-small cell lung cancer, Glioma, Pancreatic cancer, Melanoma, Gastric cancer, Apoptosis Pathways in cancer, small cell lung cancer, Proteoglycans in cancer, mTOR signaling pathway, PI3K-Akt signaling pathway

In alignment with the results of the KEGG pathway analysis, our findings highlight the shared pathways between neurodegenerative diseases and HCC. The enrichment study identified significant enrichment in pathways, including Pathways in Cancer, the PI3K-Akt signaling pathway, the mTOR signaling pathway, the TGF-β signaling pathway, and the specific HCC pathway. The cluster analysis further corroborates this observation, as illustrated in Fig. 4.

### 3.5 Promoter pattern analysis for central genes (promoter motif analysis of DEGs)

By examining unique folding regions (UFRs) spanning 1000 bp within differentially expressed genes (DEGs), we identified consistent patterns and consensus *cis*-regulatory elements (CREs). These UFRs were extracted using Ensembl BioMarts, a comprehensive data retrieval platform that covers a wide range of taxonomies. For identifying and evaluating motifs, we utilized the MEME Suite web server, which provides a robust online portal for motif analysis. The identified motifs can yield insights into features such as protein interaction domains and DNA binding sites. To further refine the motif analysis, we employed TOMTOM, a tool specifically designed for searching motif databases, to compare transcription factor (TF) motifs with those stored in established motif databases.

To gain deeper insights into potential functionalities, we comprehensively explored transcription factor (TF) motifs by associating them with GO categories, including BP, MF, and cellular component (CC). This analysis was facilitated by GOMO, a tool specifically designed for linking motifs to GO terms.

The initial step involved using the TOMTOM tool to analyze the unique folding region (UFR) sequences to identify significant motifs. The selected motifs were subsequently assessed using GOMO. Three noteworthy motifs—BICRA, ARID2, and CASP3—were identified based on their lengths. These motifs are associated with prominent transcription factor families known for binding to our key genes' promoters. Our investigation with GOMO, utilizing motifs discovered by MEME, revealed a range of intriguing biological roles, as summarized in Table 5.

The GO analysis has revealed various roles for these motifs, encompassing various biological processes and cellular locations. These include activities in the cytosol, ATP binding, the nucleolus, GTPase activity, transcriptional corepressor and activator functions, and signal transduction. Additionally, the motifs are implicated in ion binding, particularly with zinc and potassium ions, as well as in axon guidance, negative regulation of signal transduction, and chromatin binding, among other functions. Notably, chromatin binding is a significant aspect of their functionality.

The functions attributed to these gene motifs primarily focus on transcription regulation, chromatin modification, signal transduction, and various aspects of morphogenesis and development. These motifs are believed to play a crucial role in precise gene expression, modulation of signaling pathways, and developmental processes in organisms, owing to their multifaceted regulatory functions in transcription and chromatin architecture.

The GO analysis indicates that these motifs are predominantly involved in functions such as cytosolic localization, binding of ATP and potassium ions, involvement in the nucleolus, GTPase activity, transcription corepressor functions, assembly of transcription factors, transcriptional activation, negative regulation of signal transduction, zinc ion binding, axon guidance, and chromatin binding. The primary biological processes associated with these gene motifs include transcriptional control, chromatin modification, signal transduction, and development/morphogenesis.

These motifs play crucial roles in regulating gene expression, signal transduction, and developmental pathways within the body. Their integrated actions are essential for proper growth, cellular differentiation, and the modulation of responses to external stimuli. These biological processes are likely to significantly impact the development and progression of HCC, as summarized in Table 4.

**Table 4:** The MEME analysis of DEG promoters revealed conserved motifs. To find the CREs and conserved motifs, the UFRs of DEGs were examined. TOMTOM was used to analyze significant motifs in the retrieved UFR sequences. GOMO was then used to examine a subset of the motifs. There were three significant motifs found.

Gene	Sequence	Top 5 specific predictions
BICRA		CC cytosol, MF ATP binding, CC nucleolus MF GTPase activity, MF transcription corepressor activity
ARID2		CC transcription factor complex, MF transcription activator activity, BP negative regulation of signal transduction, MF zinc ion binding, MF potassium ion binding
CASP3		CC transcription factor complex, BP axon guidance, BP negative regulation of signal transduction, MF chromatin binding, BP inner ear morphogenesis

### 3.6 Expression analysis of the hub genes

**Fig. 5:**
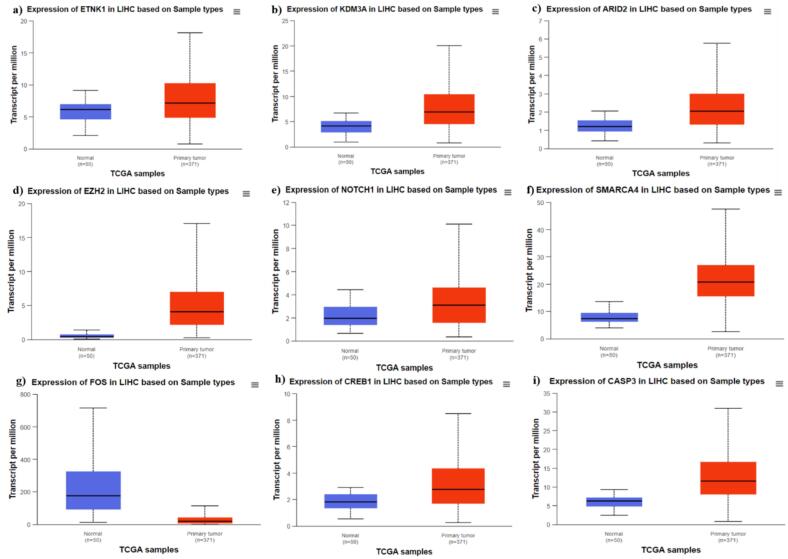
Expression analysis of the hub genes. Box plot analysis of gene expression levels in HCC and normal tissues using UALCAN data. The hub genes ETNK1, KDM3A, ARID2, EZH2, NOTCH1, SMARCA4, CREB1, and CASP3 show significant overexpression in HCC tissues, while FOS shows downregulation in tumor tissues compared to normal tissues.

The UALCAN data analysis portal was utilized to examine the expression profiles of the identified hub genes in HCC tissues compared to normal hepatocellular tissues. Fig. 5 presents box plots illustrating the expression levels of each gene in both tumor and normal samples. The analysis indicates that most hub genes, including ETNK1, KDM3A, ARID2, EZH2, NOTCH1, SMARCA4, CREB1, and CASP3, are significantly overexpressed in primary tumor tissues relative to normal tissues. This marked overexpression suggests that these genes may play crucial roles in the development and progression of HCC. Conversely, FOS exhibits a distinct pattern with reduced expression in tumor tissues compared to normal tissues, indicating a potential suppressive function in hepatocellular carcinoma. The observed differences in expression levels between tumor and normal tissues highlight the potential of these hub genes as biomarkers or therapeutic targets in HCC.

### 3.7 Survival analysis of the hub genes

**Fig. 6:**
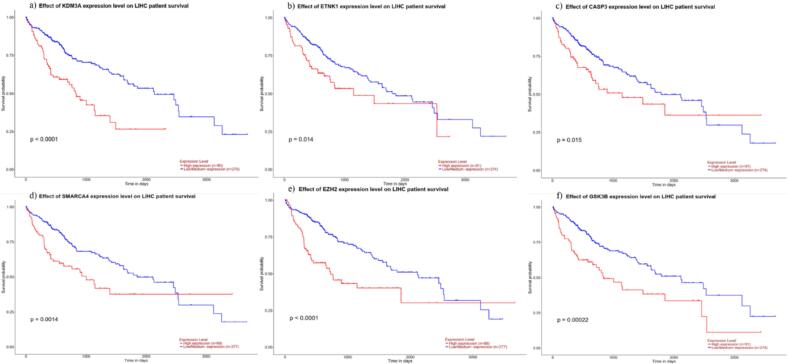
Kaplan-Meier survival analysis of hub gene expression levels in HCC patients. The plots show the overall survival of patients based on the differential expression of (a) KDM3A (p < 0.0001), (b) ETNK1 (p = 0.014), (c) CASP3 (p = 0.015), (d) SMARCA4 (p = 0.0014), (e) EZH2 (p < 0.0001), and (f) GSK3B (p = 0.00022). High expression levels of these genes are associated with poorer patient survival.

Survival analysis conducted using the UALCAN portal revealed significant associations between the expression levels of several hub genes and overall survival in patients with HCC. Fig. 6 presents the Kaplan-Meier survival curves for six hub genes: KDM3A, ETNK1, CASP3, SMARCA4, EZH2, and GSK3B. Patients exhibiting high expression levels of KDM3A demonstrated significantly poorer survival outcomes than those with low expression (p < 0.0001). Similarly, high expression of ETNK1 was associated with reduced survival (p = 0.014). Elevated levels of CASP3 correlated with worse overall survival (p = 0.015), while higher expression of SMARCA4 was linked to a significantly lower survival probability (p = 0.0014). A notable difference in survival was observed between patients with high and low EZH2 expression, with a p-value of < 0.0001, underscoring its potential as a prognostic marker. Additionally, high expression of GSK3B was strongly correlated with poor survival outcomes (p = 0.00022). These findings suggest that the overexpression of these genes may play a critical role in HCC progression and could serve as valuable prognostic markers.

### 3.8 Screening of the drugs targeting the hub genes

The drug screening analysis identified several drugs targeting key hub genes implicated in HCC (Table 5). For GSK3β, three drugs were identified: Lithium citrate (DB14507) and Lithium carbonate (DB14509), each having four known targets, including IMPA2, IMPA1, and GRIA3. Fostamatinib (DB12010) was also identified, characterized by a broad target range affecting 300 proteins, such as SYK, ADORA3, SLC18A2, and FAAH.

**Table 5:** Summary of drug screening for hub genes in hepatocellular carcinoma.

**Gene**	**Drug**	**Drug Bank ID**	**Drug Targets Number**	**Other Drug Targets**	**Structure**
**GSK3β**	Lithium citrate	DB14507	4	IMPA2, IMPA1, GRIA3	
	Lithium carbonate	DB14509	4	IMPA2, IMPA1, GRIA3	
	Fostamatinib	DB12010	300	SYK, ADORA3, SLC18A2, FAAH, etc.	
**EZH2**	Tazemetostat	DB12887	2	EZH1	
**FOS**	Nadroparin†	DB08813	4	SERPINC1, SELP, MYC	
	Nandrolone decanoate	DB08804	6	AR, HTR1B, IGF1R, Dopamine receptor, 5-hydroxytryptamine 2 receptor	
**CASP3**	Pamidronic acid	DB00282	5	FDPS, Hydroxylapatite, GGPS1, CASP9	
	Glycyrrhizic acid	DB13751	5	HSD11B1, TNF, Nuclear factor NF-kappa-B, LPL	
	Minocycline	DB01017	12	rpsI, rpsD, IL1B etc.	
	Acetylsalicylic acid	DB00945	20	PTGS1, HMGCR, PTGS2 etc.	

† Nadroparin (marketed under trade names such as Fraxiparine, Fraxodi, and others) is an anticoagulant that belongs to the class of drugs known as low molecular weight heparins (LMWHs). Heparin's general structure is shown in the table.

EZH2 is specifically targeted by Tazemetostat (DB12887), which also inhibits EZH1, underscoring its specificity and therapeutic relevance in cancer treatment. For FOS, potential drug candidates include Nadroparin (DB08813) and Nandrolone decanoate (DB08804). Nadroparin targets four proteins, including SERPINC1, SELP, and MYC, while Nandrolone decanoate interacts with six proteins, including the androgen receptor (AR), HTR1B, IGF1R, dopamine receptor, and 5-hydroxytryptamine 2 receptor.

CASP3 is targeted by several drugs, including Pamidronic acid (DB00282), which has five known targets, such as FDPS, Hydroxylapatite, GGPS1, and CASP9. Glycyrrhizic acid (DB13751) interacts with five proteins, including HSD11B1, TNF, nuclear factor NF-kappa-B, and LPL. Minocycline (DB01017) targets 12 proteins, including rpsI, rpsD, and IL1B, while Acetylsalicylic acid (DB00945) has 20 targets, including PTGS1, HMGCR, and PTGS2.

This analysis highlights several drugs that may interact with the identified hub genes, potentially providing new avenues for targeted therapeutic strategies in HCC.

## 4 Discussion

Understanding the intricate molecular network underlying HCC is crucial for developing effective therapeutic strategies. The hsa-miR-101-3p is well-established as a tumor suppressor in various cancers, including HCC. However, its precise role in HCC remains incompletely understood.^19^, ^20^ To address this knowledge gap, we conducted an extensive bioinformatics investigation to elucidate the functions of miR-101-3p and its associated targets in HCC. Our study employed a comprehensive approach to dissect the complex molecular landscape of this disease. Through our investigation, we identified critical genes and pathways associated with hsa-miR-101-3p, thereby providing valuable insights into the regulatory networks that influence the progression of HCC.

Our analysis identified twelve pivotal hub genes ETNK1, BICRA, IL-1R1, KDM3A, ARID2, GSK3β, EZH2, NOTCH1, SMARCA4, FOS, CREB1, and CASP3 that play significant roles in driving the molecular dynamics of HCC. By uncovering previously unrecognized information regarding miR-101-3p and its associated targets, our study contributes to a more comprehensive understanding of the pathogenesis of HCC. These findings have important implications for the development of novel therapeutic strategies, not only for HCC but also for other types of cancer.

Several key genes identified in this study are crucial for processes associated with cancer, including uncontrolled cell growth, evasion of programmed cell death, and initiation of tissue invasion and metastasis. These genes not only function as essential regulators within the miR-101-3p network but also serve as potential orchestrators of various cellular activities. Importantly, some of these major genes associated with miR-101-3p have potential as biomarkers for diagnosis or prognosis in HCC.

EZH2 promotes oncogenesis through epigenetic modifications. In HCC, IL-1R1 knockout reduced tumor growth, steatosis, insulin resistance, and immune cell infiltration.^21^, ^22^, ^23^ In lung cancer, IL-1β downregulated miR-101, leading to EZH2 upregulation, while miR-101 overexpression suppressed tumor growth. In medulloblastoma, exosomal miR-101-3p and miR-423-5p inhibited tumor progression by targeting FOXP4 and EZH2.^24^

GSK-3β inhibition reduced HCC cell viability and glycolysis via AMPK/mTOR signaling, making it a potential therapeutic target.^25^ In temozolomide (TMZ)-resistant glioblastoma multiforme (GBM), miR-101 downregulation led to GSK3β upregulation, promoting O6-methylguanine-DNA methyltransferase (MGMT) expression and resistance, while miR-101 overexpression sensitized cells to TMZ.^26^ In ESCC, miR-101-3p suppressed Wnt signaling and inhibited tumor progression.^27^

Notch signaling promotes HCC progression and is linked to poor prognosis.^28^ In T-ALL, miR-101 downregulation led to Notch1 upregulation, enhancing proliferation and invasion, while miR-101 overexpression suppressed Notch1 and sensitized cells to Adriamycin.^29^ In colorectal cancer (CRC), circAPLP2 sponged miR-101-3p, activating Notch1 and promoting tumor progression, while circAPLP2 knockdown inhibited tumor growth and metastasis.^30^

Upregulated cholesterol biosynthesis via SREBP2 promoted liver cancer stem cell (CSC) expansion and drug resistance, while simvastatin sensitized HCC cells to sorafenib.^31^ In melanoma, early loss of miR-101-3p led to genomic instability, while its reexpression inhibited proliferation and induced apoptosis by targeting Lamin B1, ATRX, CASP3, and PARP.^32^

SMARCA4/BRG1 was upregulated in HCC, promoting proliferation and early recurrence by facilitating S-phase entry and upregulating SMAD6.^33^ In breast cancer, miR-101 induced mild DNA damage and senescence by targeting UBE2N and SMARCA4, while severe damage reduced miR-101 expression and increased apoptosis.^34^

The CREB1/miR-922/ARID2 axis promoted liver cancer progression, with miR-922 targeting ARID2, while ARID2 overexpression counteracted malignancy.^35^ In colorectal carcinoma, miR-101 downregulation enhanced tumor growth, while its overexpression inhibited proliferation and migration by targeting CREB1.^36^

ETNK1 mutation is an early event in myeloid neoplasms, often co-occurring with ASXL1, TET2, EZH2, RUNX1, and SRSF2 mutations. It is associated with dysplasia, increased blasts, myelofibrosis, and genomic instability, making it a potential diagnostic marker and therapeutic target.^37^

BICRA, a subunit of the ncBAF complex, regulates transcription and epigenetics, processes frequently altered in cancer. Its interaction with BRD4 suggests a role in tumorigenesis, while loss-of-function mutations lead to chromatin dysregulation and genomic instability. BICRA’s involvement in epigenetic silencing may influence oncogene activation or tumor suppressor inactivation, contributing to cancer progression.^38^

KDM3A-C was upregulated in HCC, with KDM3A linked to poor survival, while KDM3B and KDM3C showed no survival impact. Genetic alterations in KDM3A-B affected prognosis, and KEGG analysis indicated their role in tumor behavior and treatment. KDM3 expression correlated with immune cell infiltration, including B cells, CD8+ T cells, and macrophages.^39^

ARID2 was downregulated in metastatic HCC, correlating with poor prognosis. It inhibited migration, invasion, and metastasis by repressing EMT through DNMT1-mediated Snail promoter methylation. Mutations disrupting its C2H2 domain abolished this function.^40^

The gene expression analysis revealed that several hub genes, such as ETNK1, KDM3A, EZH2, SMARCA4, and CASP3, were significantly overexpressed in HCC tissues, suggesting their potential roles in tumor progression and as biomarkers for HCC.

The survival analysis further emphasized the clinical relevance of these genes, as high expression levels of KDM3A, ETNK1, EZH2, SMARCA4, and GSK3B were associated with poorer patient outcomes, indicating their potential as prognostic markers. The differences in prognostic significance among the hub genes may be attributed to their distinct biological roles and interactions within oncogenic pathways. KDM3A plays a pivotal role in epigenetic regulation by demethylating H3K9me1/2, thereby activating transcription of oncogenes involved in cell proliferation and metastasis. Its overexpression in HCC tissues could amplify these oncogenic processes, leading to more aggressive tumor behavior and poorer patient outcomes, as reflected in the survival data (p < 0.0001).^41^ Similarly, EZH2 is a key catalytic component of PRC2, responsible for H3K27 trimethylation, which silences tumor suppressor genes. Elevated EZH2 expression is known to promote tumor progression by suppressing apoptosis and enhancing cell cycle progression. The strong prognostic significance of EZH2 (p < 0.0001) aligns with its role in driving aggressive tumor phenotypes and its involvement in pathways like PI3K/Akt and TGF-β.^42^ On the other hand, genes such as CASP3 and SMARCA4 also showed significant but slightly less pronounced associations with patient survival. CASP3, a crucial mediator of apoptosis, may exert dual roles depending on the cellular context. While its dysregulation can impair apoptosis, contributing to tumor survival, CASP3 activation can also lead to tumor cell death in certain conditions, potentially explaining the moderate significance (p = 0.015) observed in our study. SMARCA4 is a chromatin remodeler that influences gene expression by modifying nucleosome positioning. Its dysregulation can disrupt DNA repair mechanisms and promote tumor progression, as observed in HCC.^43^ However, its prognostic impact (p = 0.0014) may be moderated by the compensatory roles of other chromatin remodelers within the tumor microenvironment. These findings underscore the complexity of the regulatory networks in HCC and highlight the multifaceted roles of these genes in tumorigenesis.

The drug screening identified several drugs, including Tazemetostat for EZH2 and Lithium compounds for GSK3β, which could offer promising therapeutic strategies; however, further studies are required to validate their efficacy and safety in HCC treatment. While EZH2 has shown significant therapeutic potential, particularly with inhibitors like Tazemetostat, there are notable challenges in translating these findings into clinical practice. One limitation is the potential for off-target effects and drug resistance, which could arise due to compensatory mechanisms within the tumor microenvironment or mutations in the target site.^44^ Additionally, the heterogeneity of HCC poses a challenge, as patients may exhibit varying responses to EZH2 inhibitors depending on their genetic and epigenetic profiles.^22^ Furthermore, the systemic inhibition of EZH2 may impact normal cells, particularly stem cells, given its role in maintaining normal epigenetic regulation.^45^ This could lead to unintended side effects, such as hematopoietic toxicity. These challenges underscore the need for further research to identify biomarkers for patient stratification, optimize dosing regimens, and explore combination therapies to enhance efficacy and reduce resistance.

The identified drugs provide a foundation for developing targeted therapeutic strategies in HCC. Tazemetostat, which specifically targets EZH2, has already shown promise in clinical trials for other cancers, including follicular lymphoma and epithelioid sarcoma. Its inclusion in HCC trials could explore its efficacy in mitigating tumor progression associated with EZH2 overexpression.^46^ Similarly, lithium-based drugs, such as lithium citrate and lithium carbonate, which target GSK3β, hold potential due to their long-standing approval for psychiatric disorders and relatively low toxicity. Investigating their repurposing in HCC could provide a cost-effective therapeutic option, especially in combination with existing treatments.^47^ Fostamatinib, with its broad target range, could be evaluated in preclinical studies to assess its ability to inhibit oncogenic pathways in HCC, such as PI3K/Akt and TGF-β. Similarly, the potential of CASP3-targeting agents like minocycline and acetylsalicylic acid could be explored in inducing apoptosis in HCC cells, leveraging their well-documented safety profiles. Additionally, the anticoagulant Nadroparin, targeting FOS, could be tested for its ability to influence the tumor microenvironment and reduce metastasis in HCC patients. To bridge these findings with clinical applications, future studies could involve: a) in vitro and in vivo validation of the identified drugs to confirm their efficacy and specificity in HCC models, b) patient stratification strategies to identify HCC subgroups that would benefit most from these targeted therapies, based on biomarker analysis of hub gene expression, and c) combination therapy studies to evaluate the synergistic effects of these drugs with existing treatments, such as sorafenib, immune checkpoint inhibitors, or chemotherapeutic agents. These approaches could accelerate the translation of our findings into clinical trials and pave the way for precision medicine in HCC.

Future research should focus on validating the therapeutic potential of identified drugs through in vivo studies using HCC animal models and in vitro assays with patient-derived organoids to assess drug efficacy and specificity. Additionally, conducting retrospective and prospective studies with patient cohorts stratified by hub gene expression levels could provide insights into their clinical relevance and guide the design of targeted clinical trials.

## 5 Conclusion

The current study significantly advances our comprehension of HCC, a highly malignant cancer type. Leveraging advanced bioinformatics techniques, the identification of 12 crucial genes (ETNK1, BICRA, IL-1R1, KDM3A, ARID2, GSK3β, EZH2, NOTCH1, SMARCA4, FOS, CREB1, CASP3) associated with the tumor-suppressing miR-101-3p network stands out as a notable achievement. These genes interact with key oncogenic pathways, such as TGF-β, MAPK, mTOR, and PI3K/Akt, and participate in the negative regulation of signal transduction. This underscores the intricate nature of HCC pathogenesis and implies the potential for targeted therapeutic strategies. The study successfully bridges the gap between basic research and clinical application, underscoring the role of miR-101-3p in governing critical genes and pathways in HCC. This emphasizes its potential as both a biomarker and a therapeutic target. The identified hub genes exhibit diverse roles, from promoting to inhibiting tumor growth, thus illustrating the complex genetic networks underlying HCC.

Notably, the study identifies an intersection between HCC pathways and those implicated in neurodegenerative disorders, suggesting shared molecular mechanisms and unveiling new prospects for treatment and understanding. While this research provides a robust foundation, further empirical studies are warranted, particularly in validating these findings in clinical samples. Confirming the clinical relevance of these genes and pathways and exploring the dynamic interactions between miR-101-3p and its targets could uncover novel treatment strategies. This study not only deepens our understanding of HCC but also establishes a comprehensive basis for future research, aiming to improve patient prognosis through innovative targeted treatments. For validation requiring in vivo and in vitro studies using HCC mouse models and multicenter clinical trials with diverse patients. These approaches, combined with investigation of potential synergistic effects between conventional and targeted therapies, would strengthen our findings and accelerate their translation into clinical applications.

## CRediT authorship contribution statement

**Nasim Rahimi-Farsi:** Writing – original draft, Software, Methodology, Investigation, Formal analysis, Data curation, Conceptualization. **Abozar Ghorbani:** Writing – review & editing, Software, Project administration, Conceptualization. **Negar Mottaghi-Dastjerdi:** Writing – review & editing, Data curation. **Taha Shahbazi:** Writing – review & editing, Investigation, Data curation. **Fatemeh Bostanian:** Writing – review & editing, Formal analysis, Data curation. **Parvin Mohseni:** Writing – review & editing, Writing – original draft. **Fateme Yazdani:** Writing – original draft, Formal analysis, Data curation.

## Declaration of competing interest

The authors declare that they have no known competing financial interests or personal relationships that could have appeared to influence the work reported in this paper.

## Data Availability

The data supporting the conclusions of the study are all provided in the manuscript.
